# Supplementary figures and images for: Deriving household composition using population-scale electronic health record data—A reproducible methodology
Source: PLoS One. 2021 Mar 29;16(3):e0248195. doi: 10.1371/journal.pone.0248195 (PMC8007012; doi:10.1371/journal.pone.0248195)

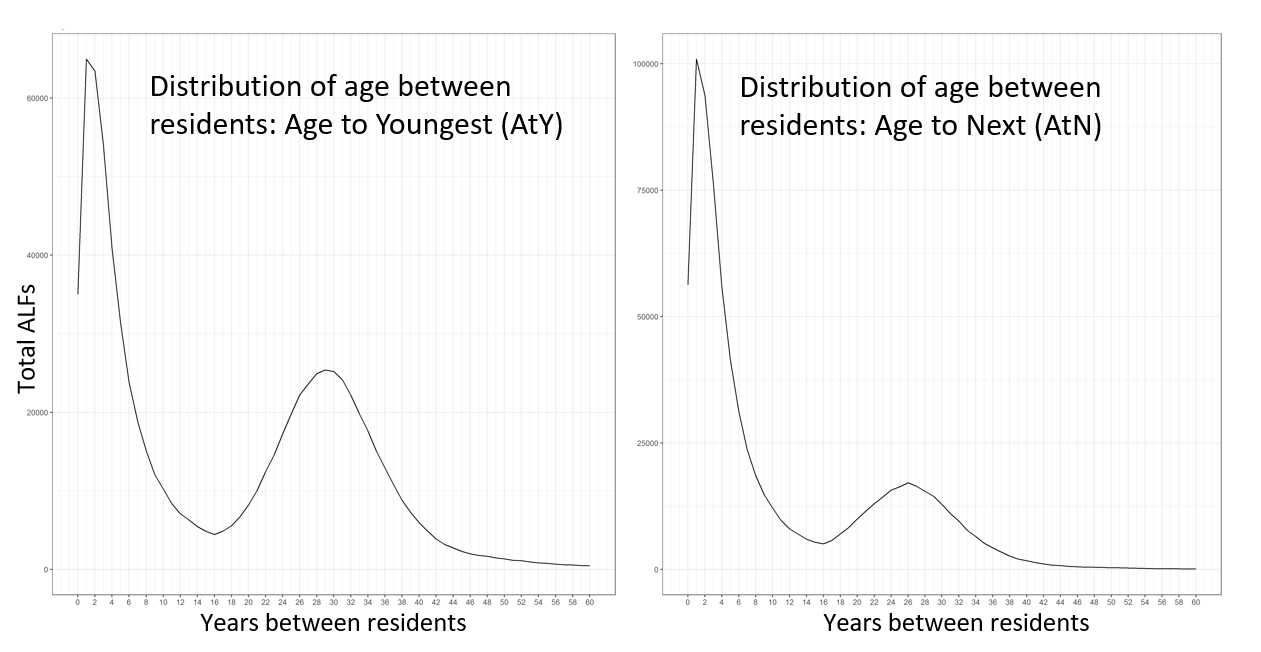

Supplement: S1 Fig — (TIF) [file pone.0248195.s001.tif]

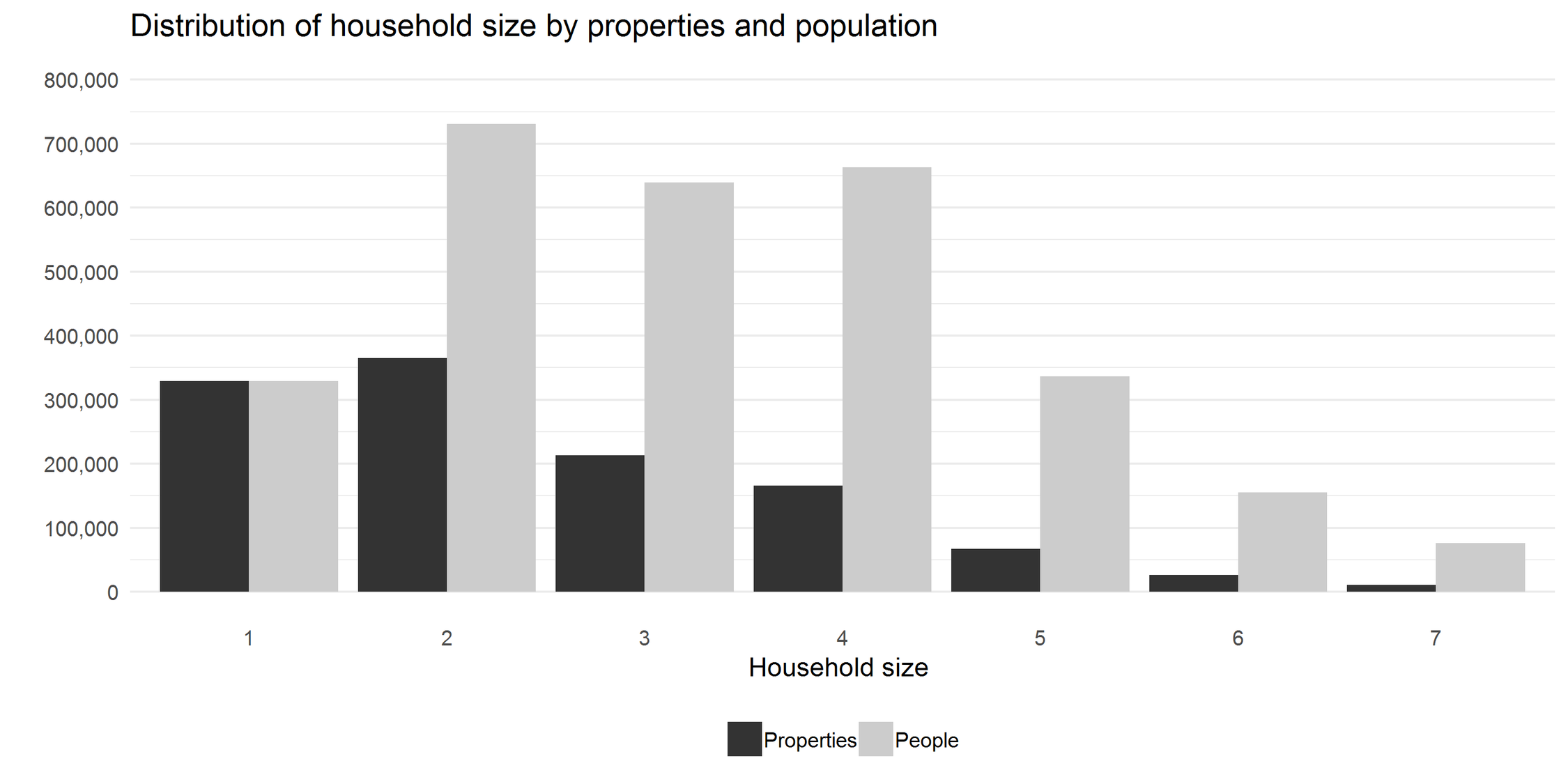

Supplement: S2 Fig — (TIF) [file pone.0248195.s002.tif]
