# Supplementary material for: Deriving household composition using population-scale electronic health record data—A reproducible methodology
Source: PLoS One. 2021 Mar 29;16(3):e0248195. doi: 10.1371/journal.pone.0248195 (PMC8007012; doi:10.1371/journal.pone.0248195)
Supplement: Attachment — Submitted filename: Response to Reviewers.docx [file pone.0248195.s003.docx]

PLOS ONE

10 February 2021

Dear Sreeram

**Re: PONE-D-20-36605**

**Deriving household composition using population-scale Electronic Health Record data – a reproducible methodology**

Thank you for the opportunity for us to revise our manuscript. We are very grateful for the very helpful reviewer suggestions and, as a result, feel that the manuscript has improved. We detail how we have addressed the comments point-by-point below.

We hope that you will now find the paper suitable for publication in PLOS ONE and look forward to hearing your decision.

yours sincerely,

Rhodri Johnson

On behalf of all authors

Journal Requirements:

1.) Please ensure that your manuscript meets PLOS ONE's style requirements, including those for file naming.

Author response:

- We have checked the requirements and have made necessary changes.
- All article images have been through the PACE software as recommended to obtain the correct file specification.

2.) Thank you for stating the following financial disclosure:

Please include your amended statements within your cover letter; we will change the online submission form on your behalf.

We have updated the funding statement as follows:

This work was supported by the Medical Research Council (MRC) [MR/V028367/1]; Health Data Research (HDR) UK [HDR-9006] which receives its funding from the UK Medical Research Council, Engineering and Physical Sciences Research Council, Economic and Social Research Council, Department of Health and Social Care (England), Chief Scientist Office of the Scottish Government Health and Social Care Directorates, Health and Social Care Research and Development Division (Welsh Government), Public Health Agency (Northern Ireland), British Heart Foundation (BHF) and the Wellcome Trust; Administrative Data Research (ADR) UK which is funded by the Economic and Social Research Council [grant ES/S007393/1]; and the Nuffield Family Justice Observatory (FJO) [FJO/43766]. At the time when the work was undertaken, the authors were supported by these funders as follows: RDJ (ADR and Nuffield FJO); LJG (ADR UK, Nuffield FJO); JH (MRC); AA (HDR UK, ADR UK, MRC), AL (MRC, NFJO), RAL (HDR UK, ADR UK, MRC), RF (HDR UK, ADR UK, MRC).

The funders had no role in study design, data collection and analysis, decision to publish, or preparation of the manuscript.

3.) We note that you have indicated that data from this study are available upon request. PLOS only allows data to be available upon request if there are legal or ethical restrictions on sharing data publicly. For more information on unacceptable data access restrictions, please see <http://journals.plos.org/plosone/s/data-availability#loc-unacceptable-data-access-restrictions>.

 In your revised cover letter, please address the following prompts:

 a) If there are ethical or legal restrictions on sharing a de-identified data set, please explain them in detail (e.g., data contain potentially sensitive information, data are owned by a third-party organization, etc.) and who has imposed them (e.g., an ethics committee). Please also provide contact information for a data access committee, ethics committee, or other institutional body to which data requests may be sent.

 b) If there are no restrictions, please upload the minimal anonymized data set necessary to replicate your study findings as either Supporting Information files or to a stable, public repository and provide us with the relevant URLs, DOIs, or accession numbers. For a list of acceptable repositories, please see http://journals.plos.org/plosone/s/data-availability#loc-recommended-repositories.

 We will update your Data Availability statement on your behalf to reflect the information you provide.

The data used in this study are available in the SAIL Databank at Swansea University, Swansea, UK. All proposals to use SAIL data are subject to review by an independent Information Governance Review Panel (IGRP). Before any data can be accessed, approval must be given by the IGRP. The IGRP gives careful consideration to each project to ensure proper and appropriate use of SAIL data. When access has been approved, it is gained through a privacy-protecting safe haven and remote access system referred to as the SAIL Gateway. SAIL has established an application process to be followed by anyone who would like to access data via SAIL https://www.saildatabank.com/application-process.

**Response to Reviewers:**

We would like to thank the reviewers for their time and respond to their queries as follows:

“The flexible approach is a valuable aspect of the work. Perhaps it would be useful to expand a little more on this. My concern with the method was the ability to time update for household composition. If the census is completed every 10 years, how does time from census date affect the measures? Perhaps family households would change in the census interval faster than other household types.”

Thank you, we are pleased that the reviewer recognised that the flexible approach is a strength of the method. We have updated the article to emphasise the flexible approach whilst also taking the opportunity to remove ambiguity and to clarify the temporal nature of the data. Our term ‘census’ relates to the date at which we ran the algorithm for the date included in the manuscript. The method does not use ONS Census data but uses individuals’ GP address registration data in the Welsh Demographic Service data set (WDSD) held in anonymised form in the SAIL Databank. Our method can therefore be used to create the relevant household measures at any date across the date range available (1990 to almost current day – researchers can request the most up to date data which is within a month of being current <https://data.ukserp.ac.uk/Asset/View/20>).

The query relating to how results may differ depending upon proximity to the census date are therefore not relevant within this context. However, as we are able to create results at any point this perhaps emphasises the flexibility of the approach and the ability to understand change over time.

We have made the following manuscript revisions:

- Replaced the phrase ‘census date’ with ‘index date’ where appropriate to emphasise that the SAIL data used is not reliant upon ONS Census data
- Within the study population section, we added: “…however, the methods can be run at any index date within the WDSD date range (early 1990’s to current day).”
- Within the summary of main findings we added: “Individuals’ anonymised address registration records are available in SAIL from the early 1990’s to current day with new data added frequently; this adds flexibility to the methods which can be run at any specific index date, or at multiple time points to obtain measures of change for longitudinal studies”.

“Were any of the age differences data driven? Family dynamics are changing over time. Could this be accounted for in long-term studies?”

The age differences selected were not data driven but were based on a pragmatic approach of fixed age generational boundaries; we mention this limitation within the manuscript alongside the need for a general model. We also provide information relating to the age differences between residents in the supplementary file (S1) which could be used to inform a data driven approach, which could also be used to inform changes in the algorithm over time.

Specific manuscript changes are as follows:

- The following phrase has been added to the end of the household composition section: “…could be used to inform future adaptations of the method to a data driven approach”.

“Related, students and their living arrangements have been an important consideration for infection control efforts. Could the authors comment on whether the data is available (or might be available) to identify households with students?”

Thank you for raising this important issue. There is currently no specific indicator of student housing available in SAIL. To mitigate against the effects of capturing student residences the current study excluded larger households by limiting to those sized seven or less; therefore, larger student accommodation would not have been captured.  However, a proxy measure could be used to infer whether a property was likely to be student accommodation. For instance, a household with a code of ‘7-0-0’ would indicate seven individuals 18 years of age of each other; combined with an indicator of age of youngest resident, or an average age, it could be inferred if the property was a student dwelling. This could further be developed using the LSOA to understand if the area was close to universities in Wales and increased chance of being a student dwelling. There would be difficulties in confidently differentiating between dwellings that may be shared residences but not students. Further work is currently underway with Welsh Government to link post 16 education data into SAIL to help further understand this population subgroup.

Specific manuscript changes are as follows:

- The following change has been made to the ‘Strengths and limitations’ section: “…The existing method could be used to infer certain dwelling types for specific research projects. For example, a household ‘AtY’ code of 7-0-0 in combination with average household age may allow student households to be selected for specific research aims”.
- The following rewording and additional change has been made to the ‘Future methodology improvements’ section: “…future developments include linkage of administrative and GIS derived data to refine housing type classifications enabling additional classification of houses in multiple occupation and communal residences (e.g. student housing and halls of residence)”.

Some more extensive footnotes for each of the figures would be useful so that they can be considered without referring back to the text.

Unfortunately, PLOS ONE guidelines state that footnotes are not allowed and such information should be added to the main or reference text, therefore we have made no changes.

We have added a further reference [38] within the ‘Existing evidence’ section:

Harper G, Mayhew L. Using Administrative Data to Count and Classify Households with Local Applications. Appl Spat Anal Policy [Internet]. 2016 Dec 11 [cited 2021 Feb 8];9(4):433–62. Available from: http://research.dwp.gov.
